# Exploration of the Rapid Effects of Personal Fine Particulate Matter Exposure on Arterial Hemodynamics and Vascular Function during the Same Day

**DOI:** 10.1289/ehp.1002107

**Published:** 2010-12-06

**Authors:** Robert D. Brook, Hwashin H. Shin, Robert L. Bard, Richard T. Burnett, Alan Vette, Carry Croghan, Jonathan Thornburg, Charles Rodes, Ron Williams

**Affiliations:** 1 Division of Cardiovascular Medicine, University of Michigan, Ann Arbor, Michigan, USA; 2 Biostatistics and Epidemiology Division, Health Canada, Ottawa, Ontario, Canada; 3 U.S. Environmental Protection Agency, Research Triangle Park, North Carolina, USA; 4 RTI International, Research Triangle Park, North Carolina, USA

**Keywords:** endothelium, heart rate, particulate matter air pollution, personal exposure monitoring, sympathetic nervous system

## Abstract

**Background:**

Levels of fine particulate matter [≤ 2.5 μm in aerodynamic diameter (PM_2.5_)] are associated with alterations in arterial hemodynamics and vascular function. However, the characteristics of the same-day exposure–response relationships remain unclear.

**Objectives:**

We aimed to explore the effects of personal PM_2.5_ exposures within the preceding 24 hr on blood pressure (BP), heart rate (HR), brachial artery diameter (BAD), endothelial function [flow-mediated dilatation (FMD)], and nitroglycerin-mediated dilatation (NMD).

**Methods:**

Fifty-one nonsmoking subjects had up to 5 consecutive days of 24-hr personal PM_2.5_ monitoring and daily cardiovascular (CV) measurements during summer and/or winter periods. The associations between integrated hour-long total personal PM_2.5_ exposure (TPE) levels (continuous nephelometry among compliant subjects with low secondhand tobacco smoke exposures; *n* = 30) with the CV outcomes were assessed over a 24-hr period by linear mixed models.

**Results:**

We observed the strongest associations (and smallest estimation errors) between HR and TPE recorded 1–10 hr before CV measurements. The associations were not pronounced for the other time lags (11–24 hr). The associations between TPE and FMD or BAD did not show as clear a temporal pattern. However, we found some suggestion of a negative association with FMD and a positive association with BAD related to TPE just before measurement (0–2 hr).

**Conclusions:**

Brief elevations in ambient TPE levels encountered during routine daily activity were associated with small increases in HR and trends toward conduit arterial vasodilatation and endothelial dysfunction within a few hours of exposure. These responses could reflect acute PM_2.5_-induced autonomic imbalance and may factor in the associated rapid increase in CV risk among susceptible individuals.

Particulate matter (PM) ≤ 2.5 μm in aerodynamic diameter (PM_2.5_) is associated with an increased risk for cardiovascular (CV) morbidity and mortality, even within the same day of exposure (Peters et al. 2001). PM_2.5_-induced endothelial dysfunction and elevated blood pressure (BP) may be playing important causative roles (Brook 2008). Indeed, higher levels of ambient PM_2.5_ have been associated with blunted brachial flow–mediated dilatation (FMD) and elevated BP (Auchincloss et al. 2008; Chuang et al. 2005; Dales et al. 2007; Dvonch et al. 2009; Ebelt et al. 2005; Langrish et al. 2009; Liu et al. 2009; O’Neill et al. 2005; Rundell et al. 2007; Schneider et al. 2008; Zanobetti et al. 2004). However, most studies have examined these outcomes only ≥ 1 days after relatively prolonged exposures (e.g., 24- to 72-hr mean PM_2.5_ levels) (Brook and Rajagopalan 2009). Whether brief (e.g., hour-long) exposures to PM_2.5_ at commonly encountered present-day ambient levels can alter arterial hemodynamics and vascular function as rapidly as within the same day and the characteristics (e.g., magnitude, consistency) of any such responses remain unclear. Because 2-hr-long exposure to very high particle levels under experimental conditions can elevate BP and impair FMD within hours (Brook et al. 2009), it is plausible that PM_2.5_ at lower ambient levels may induce similar responses.

The relationship between personal exposures and ambient PM_2.5_ mass concentrations can significantly vary among individuals, with personal levels potentially being more predictive of health responses (Brook et al. 2010; Rodes et al. 2010). Exposure characterizations at the personal level thus advance our understanding of the complex relationships between pollution exposures and diseases (Weis et al. 2005). The CV substudy of the Detroit Exposure Aerosol Research Study (DEARS) was designed to elucidate the air pollution components and time frames of exposure responsible for affecting several CV outcomes, including arterial hemodynamics [i.e., BP and heart rate (HR)] and vascular function. The initial results compared the effects associated with daily (24-hr) mean ambient PM_2.5_ exposures assessed at personal versus community levels (Brook et al. 2010). In this analysis, we aimed to gain insight into the acute same-day exposure–response relationships at a finer time-scale resolution. We explored the associations between the CV outcomes, including BP, FMD, and HR, and changes in total personal PM_2.5_ exposure (TPE) measured each hour during routine daily activity throughout the preceding 24-hr period.

## Materials and Methods

This study was approved by the institutional review boards of the University of Michigan and RTI International along with the human subjects–approving official of the U.S. Environmental Protection Agency. The main DEARS protocol has been described in more detail elsewhere (Williams 2005; Williams et al. 2009). All participants were nonsmokers living in a nonsmoking household, at least 18 years old, ambulatory, and capable of understanding the consent documentation. There were no exclusion criteria for race, occupation, sex, medications, or health status. In addition to community and residential PM_2.5_ site monitoring that occurred within six Detroit, Michigan, area neighborhoods during summer and winter periods over 3 years (six total seasons), TPE was also measured for 5 consecutive days during winter and/or summer months using personal vest monitors for one study volunteer from each participating household. DEARS exposure study participants were invited without any restrictions to also participate in the CV substudy while it was occurring (during seasons 2–6). Volunteers crossing over into the CV study underwent an additional visit, when written informed consent was obtained and the average of the second and third of three seated BP measurements was determined along with a fasting lipid profile and glucose (Cholestech LDX analyzer; Cholestech Corp., Hayward, CA, USA).

### Exposure assessments

The primary PM_2.5_ personal monitoring reported here was performed using a modified personal DataRam nephelometer (model pDR-1200; Thermo Electron, Franklin, MA, USA). These devices are capable of the near-continuous active sampling and analysis of PM in either an active or passive sampling mode. The device was operated in an active mode (30-sec data collection intervals) for DEARS so that a true size fractionation of airborne PM aerosol could be estimated over the course of each 24-hr monitoring period. Modification of the basic unit consisted of installation of an active PM_2.5_ personal environmental monitor (PEM) size-fractionating inlet upstream of the optical chamber. Williams et al. (2009) have previously described in detail the use and modification of this monitor in DEARS. The basic personal nephelometer deployed here has been used to provide thousands of short-interval PM mass concentration measurements in human exposure longitudinal panel studies. We have previously reported on the operation and utility of the monitor for such purposes (Wallace et al. 2006) and their general comparability with filter-based (gravimetric) measurements (Rea et al. 2001). He et al. (2010) have recently reported the successful use of a similar version of this personal nephelometer in characterizing PM_2.5_ influence of HR variability alterations in an adult cohort.

The personal nephelometer was one of a number of monitoring devices incorporated into an exposure monitoring vest that each study participant wore throughout each day. PM_2.5_ nephelometry was initiated on each monitoring day (Tuesday through Saturday) at a consistent time (0900 hours ± 2.5 hr). Each monitoring session represented a continuous 24-hr period of air collection using a lightweight nylon monitoring vest to secure the sampler’s inlet in the breathing zone of the participant (Williams et al. 2003, 2009). Participants were instructed to wear the vest at all times except for periods of sleeping, bathing, and changing clothes, at which times the monitor was directed to be kept as close as possible to the subject (e.g., next to the bed at night). Quality of the nephelometric data collected in the study was ensured through rigorous quality assurance/quality control (QA/QC) procedures. In brief, each nephelometer was chamber calibrated immediately before each DEARS monitoring season using a challenge aerosol of PM_2.5_ size-fractionated ammonium nitrate, which was known to make up a sizable component of the Detroit air shed. In addition, each unit was audited once during the season for any response changes in its calibration as well as at the end of the 7-week study period. Daily audits of monitor flow rate and battery condition, among other parameters, were performed. Audits of the units’ zero concentration response point (baseline) were performed using a HEPA-grade airstream. Study data were reviewed each day for completeness (length of successful monitor operation), impact of relative humidity (RH) on monitor response, and overall data acceptability criteria (monitor response relative to changes in minute by minute data values). Wallace et al. (2006) have described in detail the procedures used here to determine overall data acceptance criteria. Because nephelometric response is known to be RH sensitive, continuous RH measurements were taken simultaneously (Williams et al. 2009). These RH were then used to establish an algorithm to correct nephelometric response for such bias. A full description of this algorithm will be reported elsewhere. All data reported here were RH corrected.

The monitoring vest deployed here contained continuous sensors that monitored how compliant participants were with wearing it during the nonexclusion scenarios or time of day (e.g., sleeping, bathing). These sensors collected information on physical activity levels and body temperature among other parameters of the participant. These electronic data were then cross-checked with 15-min interval time–activity diaries completed each day by the participant to note periods of reported sleeping, bathing, or performing other activities resulting in the monitoring equipment not being worn. Findings from this review resulted in a level of general monitoring compliance for each monitoring period. Only monitoring data from participants meeting a prespecified conservative compliance rate of 60% were analyzed in this study, for reasons described elsewhere (Rodes et al. 2010; Williams et al. 2009).

Although all participants recruited into DEARS were self-reported to be nonsmokers living in nonsmoking households, each participant’s exposure to secondhand smoke (SHS) was also measured simultaneously with the nephelometric monitoring. These data were collected using another collocated PM_2.5_ PEM inlet also affixed to the monitoring vest. This inlet contained a 37-mm Teflon filter (Teflo; Gelman Science, Ann Arbor, MI, USA) for active (2 L/min) exposure monitoring. The subsequent filter samples were optically analyzed for a mass-based estimate of SHS using a technique previously described (Lawless et al. 2004; Rodes et al. 2010; Williams et al. 2009). Only the results from subjects with a predetermined rate of < 1.5 μg/m^3^ of mean daily SHS exposure were included in this analysis to avoid its potential confounding effect on the CV outcomes as described elsewhere (Brook et al. 2010). Rodes et al. (2010) has provided a detailed examination of potential SHS impact on personal exposures in the DEARS; the threshold of 1.5 μg/m^3^ was employed to note such influence. The SHS threshold value used here results in a very conservative data inclusion criteria.

The filter sample described above also provided the means to establish a filter-based gravimetric estimate of personal PM_2.5_ exposure that could be compared with the continuous nephelometric response. This filter-based sampling technique and the actual monitoring device used here have been used to collect thousands of personal exposure samples. It provided for a 24-hr integrated estimate of PM_2.5_ personal exposure in DEARS, as described elsewhere (Brook et al. 2010; Rodes et al. 2010; Williams et al. 2009). Data from collocated personal monitoring of both samplers from each personal monitoring event were then used poststudy to determine the level of agreement between the two techniques (nephelometry and gravimetric). Nephelometric mass concentrations (micrograms per cubic meter) were highly correlated with those from the filter-based sampler. Integration of the personal nephelometric data from each 24-hr monitoring period provided a single average concentration, which in turn was compared with the filter-based monitoring, resulting in a mean coefficient of determination of *R*^2^ = 0.80 (range, 0.75–0.85) over the three DEARS monitoring periods (seasons 4–6) reported here. This level of agreement was believed to indicate the high degree of QA/QC that was employed for the study, the use of the same size-fractioning inlet for both monitoring types, the use of a calibration aerosol (ammonium nitrate) similar to that encountered in Detroit, and correcting for RH bias among other factors.

All of the PM_2.5_ nephelometric data reported here represent TPE. No source categorization of the aerosol into its various components (e.g., PM of ambient origin, PM of nonambient origin) is possible at this time. Nonambient sources would potentially include cooking aerosols and mobile source emissions, among others. Such sources often contribute 50% or more of the TPE (Wallace et al. 2006). Future efforts will attempt to integrate observational survey data collected in DEARS and determine individual sources of short duration affecting the nephelometric response.

### CV end points

CV study visits were performed at the participant’s home for up to 5 consecutive evenings, Tuesday through Saturday, between 1600 and 1900 hours. These visits took place on concurrent days while subjects wore the vest monitors. There were six CV outcomes: systolic and diastolic BP, HR, brachial artery diameter (BAD; indicative of basal arterial tone), FMD, and nitroglycerin-mediated dilatation (NMD; indicative of smooth muscle function). Participants were instructed to maintain their daily routine, including taking all medications, but to fast for at least 4 hr before the scheduled visits and to avoid unusual physical activity. During each visit, subjects rested supine for 10 min before automated BP and HR measurement (Omron 780 monitor; Omron Inc, Kyoto, Japan) that was obtained in triplicate with a 1-min lapse between measures. The average of the second and third BP and HR recordings was used for analyses (Pickering et al. 2008).

BAD and FMD were next determined. Brachial images were obtained with a portable Terason 2000 ultrasound system with a 10.0-mHz linear array transducer with electrocardiogram-gated image acquisition (http://www.terason.com/; Teratech Corp., Burlington, MA, USA). Five minutes of upper arm occlusion using a rapidly deflating arm cuff was performed in order to determine FMD, which was defined as the mean percent increase in BAD above baseline diameter from between 50 and 70 sec after cuff deflation. Images were analyzed using semiautomated edge detection software (Vascular Research Tools; Medical Imaging Applications, LLC, Coralville, IA, USA). NMD was next determined as the percent dilatation of the BAD 3 min after 0.4 mg of sublingual nitroglycerin. Detailed descriptions of the methods have been previously described and accord with guidelines (Brook et al. 2005), and the reproducibility of our testing is reported elsewhere (Brook et al. 2009). Compared with the brachial FMD study technique performed in our controlled laboratory setting as described elsewhere, the only substantive methodological differences in this study are the lack of room temperature standardization and the fact that subjects fasted for only at least 4 hr before measurements.

### Statistical assumptions and models

Integrated hour-long total PM_2.5_ exposures (i.e., TPE) during the preceding 24-hr period, calculated for each individual starting immediately before the time of their CV outcome measurements, were determined from the vest continuous nephelometry data (i.e., 24 individual hour-long periods from lag 0 to lag 23 hr), with lag 0 representing the time between 0 and 60 min before the CV outcome measurement. Two subjects with three observation periods were removed from analyses because > 25% of their hourly TPE levels during a day were negative (i.e., < 0 μg/m^3^), indicative of a systematic error in the vest monitoring system on that day, per study protocol. The associations for each hour-long TPE period were made for six prespecified outcome variables: systolic BP, diastolic BP, HR, BAD, FMD, and NMD. All of these CV outcomes were observed for 5 consecutive days for each season (winter vs. summer) within a subject.

The subjects were considered to be selected at random from a population of preselected neighborhoods per the design and methods of the main DEARS cohort, as described in more detail elsewhere (Williams 2005; Williams et al. 2009). Considering the possibility that within-subject errors were autocorrelated, a linear mixed model was employed because it was more appropriate when data were collected over time on the same subjects (Shoukri and Chaudhary 2007; West et al. 2007). We thus assumed that the association between each of the responses evaluated and exposure to TPE is linear with an intercept varying at random over individuals, but the slope was assumed to be the same for all subjects. Several predictors of the response were included in the model as fixed effects: age, sex, race, body mass index (BMI), and ambient community-level temperature (Temp). The relationship between these predictors and responses was assumed to be common to all subjects:


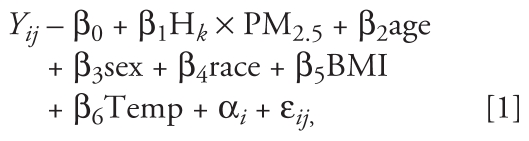


where *Y_ij_* is the response, a CV outcome for subject *i* at study day *j*, and β_1_H*_k_* × PM_2.5_ is the *k*-hr lag TPE before the health outcome measurement time. This model includes fixed effects associated with the subject-level covariates (β-values), a random effect associated with the intercept for each subject (α*_i_*), and a residual associated with each observation (ɛ*_ij_*). The random effects by subject were assumed to be independently distributed across subjects with a normal distribution α*_i_* ~ *N*(0, δ^2^). The within-subjects errors ɛ*_ij_* were assumed to be distributed ɛ*_ij_* ~ *N*(0, σ^2^*R_i_*), where *R_i_* was the variance–covariance matrix for the residuals. We also assumed that α*_i_* and ɛ*_ij_* were independent of each other.

The first-order autoregressive structure, denoted by AR(1), was explored for the covariance *R_i_* in the analysis, which implies that observations closer to each other in time exhibit higher correlation than do observations farther apart in time. The likelihood ratio test showed the AR(1) correlation structure did not improve the fit. Other available covariates, including season (i.e., winter vs. summer), neighborhood indictor, and the subject’s study day (e.g., first vs. second day of monitoring during the 5-day period), were not included in the final model because they did not predict responses individually or alter the significance of any results. We could examine only a limited number of time periods simultaneously because of the relatively small number of repeated responses per subject (maximum of five per season). Complex distributed time lag models beyond those incorporating a few lag periods were therefore not analyzed, and also because of highly correlated dependent and independent variables were inappropriate models. Although the effects of several different moving averages of exposure duration were explored, a complete description of the time course of personal PM_2.5_ exposure was most thoroughly examined for the purposes of this exploratory analysis by using 24 multiple models, each with a single hourly time lag exposure measure. The analysis was performed by function “lme” (linear mixed-effects model) in R (version 2.8.1; R Project for Statistical Computing, Vienna, Austria). Statistical significance was defined as *p* < 0.05.

## Results

The characteristics of the total cohort (*n* = 51) of subjects enrolled during seasons 4–6 who had both the CV outcomes performed and TPE measured by continuous nephelometry are presented in Supplemental Material, Tables 1 and 2 (doi:10.1289/ehp.1002107). Twenty-six subjects (51%) had no self-reported CV disease or risk factor. Ten subjects participated in two separate consecutive seasons; thus, there were a total of 61 subject-observation periods. Because subjects could contribute up to 5 consecutive days of data during each season, there were a total of 265 observation days. We excluded 74 (28%) and 91 (34%) observations by the vest compliance and low SHS rule, respectively. There were 38 subjects (191 observations) who met the 60% vest compliance rule only and 30 (102 observations) who also met the low SHS rule (vest–low SHS subgroup). Table 1 lists the characteristics of the vest–low SHS subgroup from which the main outcomes of this study are derived. The results from the vest-compliant group were similar to those from the vest–low SHS subgroup for the BAD and FMD outcomes (2-hr lag time) but not for HR changes (less significant results). Therefore, because of potential confounding effects of SHS, we proved the main results (see “Materials and Methods”) for the vest–low SHS group (Table 1).

**Figure 1:**
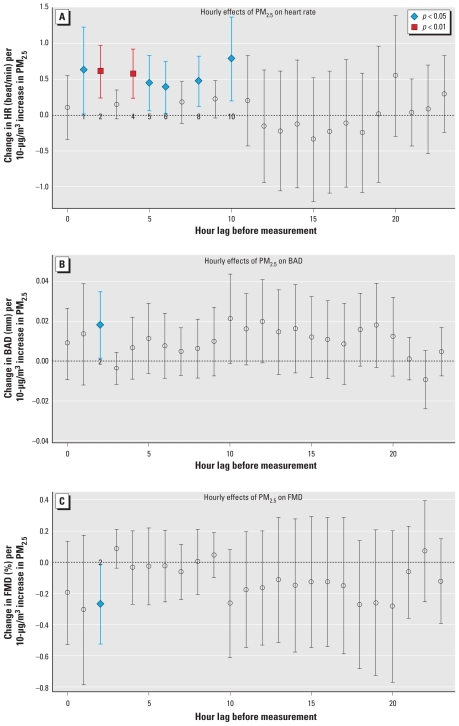
Associations of hourly TPE levels with CV outcomes according to the lag period of exposure: personal PM_2.5_ and HR (*A*), BAD (*B*), and FMD (*C*). The *x*-axis is the period of time (hour lag) before the measurement of the CV outcome; hour 0 = period from 0–60 min before the CV measurement. Points indicate the multivariate-adjusted CV outcome association (β-coefficient per 10-μg/m^3^ increase in TPE ± 95% confidence intervals) for each hourly time point from the linear mixed model 1. Colored data points indicate statistically significant time points.

The changes in the CV parameters per 10 μg/m^3^ TPE are presented by lag time in hours (0–23 hr) in Figure 1 for HR, BAD, and FMD. It is difficult to interpret the true statistical significance of each time lag because of the many lags examined. However, we did observe some tendencies in the temporal pattern. We observed positive associations for time lags of 1–10 hr for HR (0.38 to 0.78 beats/min per 10 μg/m^3^) but not for the 11–23 hr lags. We also estimated the 1–10 hr time lag effects with more precision (i.e., narrower confidence intervals) compared with the longer time lags. The temporal pattern between TPE and either BAD and FMD was neither as clear nor consistent. However, we observed an overall trend for positive and negative associations for BAD and FMD, respectively, during the 24-hr period. The strongest evidence for an association was at the 2-hr time lag for both FMD (negative association) and BAD (positive association). We observed no consistent relationships with TPE (equal numbers of positive and negative associations) for the BP levels or for NMD [see Supplemental Material, Figures 1–3 (doi:10.1289/ehp.1002107)].

Adding additional available variables to the model (e.g., season, visit day) did not affect the results. Although the general trends remained similar, evaluating the associations with longer moving averages (2–6 hr) did not provide further insights into the nature of the TPE–response relationships beyond the more complete information gained by examining the effect of each hour individually (data not shown). In this context, the mean TPE integrated over the entire preceding 24-hr period was significantly associated only with an increase in HR (0.78 beats/min per 10 μg/m^3^, *p* < 0.05). We also examined the interaction effects between TPE and medications in two ways: use of a beta blocker and use of any of the five CV medications reported [see Supplemental Material, Table 2 (doi:10.1289/ehp.1002107)]. Because only three subjects in the vest–low SHS subgroup were using a beta blocker, we did not evaluate the effects in these subjects alone. On the other hand, the association between TPE and CV outcomes remained almost the same, with no changes in significance, after excluding these three subjects. Six subjects were using any medication. The medication interaction effects for HR, BAD, and FMD were insignificant, and thus the main association between TPE and the three CV outcomes did not change in significance. Finally, we observed no consistent findings related to subjects’ health status (BMI, initial HR or BP, age) modifying the outcome associations. Subjects with low CV risk (Framingham risk score below the mean) had positive HR associations similar to those of the total cohort (56 observations). Fewer subjects (27 observations) had risk above the mean, and thus the associations were not significant. The results are of unclear validity given the limited sample size but do not generally suggest that higher-risk patients have larger CV responses (or are principally responsible for the findings).

## Discussion

PM_2.5_ exposure has been shown to be capable of increasing BP/HR and impairing vascular function. However, these responses have typically been observed to occur ≥ 1 day after exposures that are ≥ 24 hr in duration and as estimated by ambient community levels (Brook and Rajagopalan 2009). This is the first study to report the BP and HR changes together with the vascular responses associated with brief personal PM_2.5_ exposures as they change each hour throughout the preceding day during routine activity. Higher TPE levels (without source considerations) encountered during several periods (most consistently during the most recent 11 hr) were related to small increases in HR. In addition, we observed concomitant early (lag hour 2) trends toward conduit artery vasodilatation (increased BAD) and impairment in endothelial function (decreased FMD). Although these effects were small in size and intermittently statistically significant, they did occur in a consistent and coherent biological manner. We acknowledge that this study was exploratory and should be considered hypothesis generating. Although chance statistical associations cannot be excluded given the numerous observations evaluated, and although the health significance must remain speculative, these responses (along with the underlying physiological pathway likely responsible) could help to explain the mechanism underlying PM_2.5_-mediated CV events that occur during the same day of exposure among susceptible individuals (Peters et al. 2001).

Few studies have investigated the effects of PM exposures within the same day on BP, HR, or vascular function (Chuang et al. 2005; Dales et al. 2007; He et al. 2010; Langrish et al. 2009; Rundell et al. 2007). In the most similar previous study, both BP and HR increased in relation to higher personal ultrafine particle levels encountered during the previous 1–2 hr among 10 patients with lung disease (Chuang et al. 2005). However, the investigators did not examine vascular function, periods of exposure before 4 hr, or the effects of PM_2.5_. The study was small and restricted to patients with lung disease; therefore, its pertinence to the general population is questionable. Four other studies have investigated the acute effect of brief ambient estimates of PM_2.5_ exposures (30–120 min) within the same day on similar CV parameters. However, in each report, except for that of He et al. (2010; see below), exposures were generated by artificial scenarios that conveyed higher concentrations than routinely encountered and with the responses evaluated only once within minutes thereafter. PM has been shown to impair FMD without affecting BP or HR at a bus stop (PM_2.5_ ~ 40 μg/m^3^) (Dales et al. 2007), to raise BP with a trend toward an increase in HR while walking next to city streets in Beijing (PM_2.5_ ~ 86–140 μg/m^3^) (Langrish et al. 2009), and to cause a decrease in BAD and FMD immediately after exercising close to a roadway (ultrafine counts ~ 115,000–134,000 particles/cm^3^) (Rundell et al. 2007). Despite variations in specific findings (possibly due to multiple methodology differences), these studies generally support our findings and the hypothesis that PM could pose an immediate threat to the CV system within hours of exposure. Our results significantly extend these reports by more fully characterizing the temporal relationships between several concomitantly measured CV outcomes with brief personal-level PM_2.5_ exposures encountered throughout the preceding full 24-hr period at more typical present-day ambient levels. Two other studies corroborate our finding that a more prolonged exposure (previous 24-hr-long mean personal PM_2.5_ level) can cause a small increase in HR around 0.44–1.15 beats/min (Liu et al. 2009; Mar et al. 2005).

In contrast to HR, we did not observe a coherent effect of same-day TPE levels on BP. This differs from some of our previous findings. Higher community PM_2.5_ levels measured 2–5 days earlier in three Detroit areas have been associated with elevations in BP (Dvonch et al. 2009). Integrated 24-hr TPE levels measured by filter-based methods from subjects participating in this CV substudy of DEARS averaged from approximately 8 to 32 hr before the time of BP measurement were also associated with an increase in systolic BP (1.4 mmHg per 10-μg/m^3^ increase) (Brook et al. 2010). Two other previous studies have reported a same-day effect of comparatively higher levels of particle exposures on BP (Chuang et al. 2005; Langrish et al. 2009) whereas another did not (Dales et al. 2007). Differences in pollutant concentrations, characteristics, constituents, or subject susceptibilities may have been responsible for these discordant findings among studies. However, the BP elevations we twice previously observed in relation to ambient PM_2.5_ levels in the same region (Detroit communities) occurred only in a delayed fashion. We hypothesize that, given the multitude of physiological parameters regulating systemic arterial BP, these relatively low TPE concentrations may require a longer cumulative duration of exposure and/or lag period in order to elicit an observable effect (Brook and Rajagopalan, 2009). On the other hand, the biological pathway responsible for causing an increase in HR may be more acutely sensitive to these low-level PM_2.5_ concentrations, or alternatively, small changes in chronotropic responses may be a more statistically discernible outcome.

### Potential biological mechanisms

This study was not designed to elucidate the mechanisms responsible for the observed CV changes. We acknowledge that any such discussion is speculative. However, we believe that the sum results can be interpreted to provide an overall hypothesis. There are three general pathways that could link PM_2.5_ exposure with changes in CV physiology: systemic inflammation (pathway 1), altered autonomic nervous system balance (pathway 2), and direct effects of particles or constituents reaching the circulation (pathway 3) (Brook 2008). It cannot be excluded that each pathway alone and/or as an integrated response altogether was responsible for the seemingly mixed physiological changes observed in HR, FMD, and BAD. However, given the rapidity of the responses, it is probable that pathways 2 or 3 were chiefly involved. Although effects via pathway 3 cannot be excluded, given the low concentrations of PM_2.5_ it seems most plausible to principally instigate pathway 2. Thus, we believe that acute vagal withdrawal with a (relative) increase in sympathetic nervous system activity likely caused the elevation in HR.

It is also possible that pathway 2 alone could have caused the observed trends toward conduit artery vasodilatation and blunted endothelial function within this rapid time frame. There is complex interaction between the autonomic nervous system and vascular tone/endothelial function (Harris and Matthews 2004). Acute sympathetically mediated α-receptor–induced vasoconstriction in the resistance arterioles is regionally discordant, occurring in mesenteric but not muscle beds (i.e., brachial artery) upon sympathetic stimulation. In addition, a vasoconstrictive response is blunted by a reflex increase in basal nitric oxide release in conduit vessels supplying skeletal muscles and by β_2_ receptor activation that cause vasodilatation (i.e., increased BAD), allowing for adequate muscle blood flow under periods of stress. Other studies also demonstrate that sympathetic activation subsequently leads to a rapid impairment within hours in stimulated (e.g., flow-mediated) brachial artery endothelial-dependent vasodilatation (Hijmering et al. 2002). Thus, all three outcomes observed in this study may have stemmed from a rapid imbalance of autonomic nervous system activity. A recent study by He et al. (2010) supports this speculation. He et al. (2010) similarly found that HR increased rapidly 3–6 hr after elevations in personal-level PM_2.5_ exposure. Moreover, HR variability metrics suggestive of autonomic imbalance favoring sympathetic tone occurred during this same rapid time window.

### Strengths and limitations

The CV substudy of DEARS is the first to investigate the effect of air pollutants on these CV outcomes measured in the actual “field” (i.e., households of subjects). This methodology avoided the atypical exposures and activities that would occur when subjects travel to a research laboratory and thereby strengthened our ability to observe the “real” exposure–response associations occurring on typical days. Conducting personal-level PM_2.5_ exposure assessments and adjusting for the confounding effects of vest compliance and SHS also minimized the level of exposure misclassification (Rodes et al. 2010). Such steps also improved the data quality and the proper characterization of those most exposed at the individual level, which in turn improved the power of the data content at all levels of the exposure distributions. These procedures strengthened our capabilities to observe small health effects, as reported earlier (Brook et al. 2010), within a relatively small sample size and theoretically added support for the veracity of the health–exposure associations. These robust methodologies are critically important in order to establish exposure-to-outcome linkages, particularly given that the composition and character of the PM_2.5_ can differ across the exposure distribution (Edwards and Jantunen 2009).

We also recognize several limitations. We made no attempt to examine the personal exposure factors or time–activity patterns of the participants and their potential impact on the health outcomes. Future investigations are planned that will determine the ambient and major nonambient sources encountered by participants in various locations as well as their impact on significant health outcomes. The study was also not designed to assess the associations between hourly TPE levels and the CV outcomes. The exploratory nature and multiple comparisons performed in this analysis make it possible that some of the significant results represent chance findings. However, we believe that the consistent effects on HR within the first 11 hr and the coherency of the trends of effects for BAD and FMD throughout the monitoring period suggest that true biological responses are likely occurring. It is possible that having more observations would have provided additional power and that many of the borderline non-significant time points in the BAD and FMD trends would have become statistically significant. Future appropriately designed and powered studies are required to corroborate these novel findings. The ultrasound measurements were also performed at the subject’s household. Although standardized methodologies and subject preparation were carefully followed (Brook et al. 2005), the reproducibility of the FMD and BAD may have been less than what can be obtained in a vascular laboratory. However, we believe that the overall strengths of this design (i.e., performing the studies in the actual “field”) outweighed this limitation. The statistical significance of the small changes observed for HR, BAD, and FMD suggests that the sample size using these methods was adequate. Finally, regarding the vascular responses, we observed a trend toward an increase in the basal BAD during the time period analyzed. A larger initial BAD could mathematically produce a smaller FMD response when the latter is expressed as a percent dilatation from baseline. We cannot exclude that this may have contributed to a decrease in FMD (percentage), and future studies should therefore analyze both the percent and absolute (in millimeters) vasodilatory responses when basal diameter (i.e., resting arterial tone) may be altered.

Although the main DEARS protocol sampled a random representation of the local residents, the findings from this substudy may not generalize to the entire population. However, compared with previous similar studies, the participants in this analysis represented a relatively larger cohort of individuals with a broader range of health conditions. Although we performed exploratory analyses regarding potential effect modifiers (e.g., medications, demographics), further investigation related to the effects of patient susceptibilities is warranted. Finally, the responsible PM_2.5_ components and sources could not be determined from these data. We also did not have hourly personal-level gaseous pollutant information, and there is a possibility for unrecognized additional, or confounding, effects of these copollutants. Ongoing and planned analyses may help provide future insights into these important matters.

## Conclusions

Higher TPE levels encountered during routine daily activity were related to small increases in HR and trends toward endothelial dysfunction within hours of exposure among individuals living in several Detroit-area communities. Although the clinical relevance of these responses remains unknown, the findings support in general the notion that present-day levels of PM_2.5_ could potentially rapidly affect CV physiology in a manner contributing to the instigation of acute CV events in susceptible people.

## Figures and Tables

**Table 1:** Subject characteristics in the vest compliance and low SHS subgroup (*n* = 30).

Factor	No. of observations	Mean ± SD	Minimum	Q1	Median	Q3	Maximum
Age (years)	30	45.4 ± 14.3	22	33	46	54	73
Sex (%)	30						
Female	25	83					
Male	5	17					
Race (%)	30						
African American	13	43					
Caucasian	16	53					
American Indian	1	3					
BMI (kg/m^2^)	30	29.9 ± 5.9	21.8	25.3	29.9	33.0	48.2
SBP (mmHg)	100	123.0 ± 16.5	91	110	124	136	167
DBP (mmHg)	100	73.5 ± 10.4	53	65	73	81	101
HR (beats/min)	100	74.0 ± 10.2	51	67	74	79	100
BAD (mm)	98	4.0 ± 0.9	2.5	3.4	3.9	4.6	6.2
FMD (%)	93	4.0 ± 5.2	−6.0	0.4	2.9	6.9	18.3
NMD (%)	47	15.2 ± 7.0	1.5	9.9	15.4	19.0	31.9
Average of 24 hourly PM_2.5_ (μg/m^3^)	98	32.1 ± 26.2	2.5	13.1	24.3	43.1	122.2
Daily average of personal PM_2.5_ (μg/m^3^)	102	18.0 ± 10.4	1.3	9.9	15.8	23.4	51.9
Daily average of ambient PM_2.5_ (μg/m^3^)	97	15.8 ± 7.6	5.9	9.9	13.0	20.4	38.9

Abbreviations: DBP, diastolic BP; Q1, 25th percentile; Q3, 75th percentile; SBP, systolic BP. Values are mean ± SD except where noted.
